# NetControl4BioMed: a pipeline for biomedical data acquisition and analysis of network controllability

**DOI:** 10.1186/s12859-018-2177-3

**Published:** 2018-07-09

**Authors:** Krishna Kanhaiya, Vladimir Rogojin, Keivan Kazemi, Eugen Czeizler, Ion Petre

**Affiliations:** 1 Computational Biomodeling Laboratory, Turku Centre for Computer Science, and Department of Computer Science, Å bo Akademi University, Domkyrkotorget 3, Turku, 20500 Finland; 2 National Institute for Research and Development for Biological Sciences, Splaiul Independentei 296, Bucharest, 060031 Romania

**Keywords:** Network controllability, Software pipeline, Web service, Data acquisition and integration, Protein-protein interaction networks, Personalized medicine, Cancer

## Abstract

**Background:**

Network controllability focuses on discovering combinations of external interventions that can drive a biological system to a desired configuration. In practice, this approach translates into finding a combined multi-drug therapy in order to induce a desired response from a cell; this can lead to developments of novel therapeutic approaches for systemic diseases like cancer.

**Result:**

We develop a novel bioinformatics data analysis pipeline called *NetControl4BioMed* based on the concept of target structural control of linear networks. Our pipeline generates novel molecular interaction networks by combining pathway data from various public databases starting from the user’s query. The pipeline then identifies a set of nodes that is enough to control a given, user-defined set of *disease-specific essential proteins* in the network, i.e., it is able to induce a change in their configuration from any initial state to any final state. We provide both the source code of the pipeline as well as an online web-service based on this pipeline http://combio.abo.fi/nc/net_control/remote_call.php.

**Conclusion:**

The pipeline can be used by researchers for controlling and better understanding of molecular interaction networks through combinatorial multi-drug therapies, for more efficient therapeutic approaches and personalised medicine.

**Electronic supplementary material:**

The online version of this article (10.1186/s12859-018-2177-3) contains supplementary material, which is available to authorized users.

## Background

Over the last decade, high-throughput experimental technologies like gene sequencing, proteomics, etc. became the core of biomedical research and have generated a large set of biomedical data [1]. The recent advances in experimental data acquisitions allow researchers to study functions and properties of proteins, RNAs and genes, as well as to explore a network of interactions between them. The signal transduction network of protein-protein interactions (*PPIs*) is the backbone of signalling pathways [2], metabolic pathways [3], and various essential cell processes for normal cell function [4, 5]. Such networks are modelled mathematically as directed graphs, consisting of nodes standing for all the proteins in the network, and directed edges between them standing for each signal transduction relationship between them. Each edge carries a positive “weight” signifying the relative strength of the corresponding interaction. One may associate to nodes variables that follow the dynamic level of the protein corresponding to that node. Each variable is influenced through its incoming edges by the level of its predecessors in the network, and it influences itself through its outgoing edges the level of all its successors in the network. The quantitative level of this influence is usually described through a computational model based on difference equations or ordinary differential equations. The result is a *linear dynamic system* where changes in some variable cascade through the network eventually influencing the levels of many nodes in the network. We call *configuration* or *state* (at some given time point) the collection of the levels of all variables associated to nodes in the network (at that time point).

In recent years, analysis of such directed signalling PPI networks through linear dynamical systems has been central for the current biological research, providing novel insights into modern molecular biology from the network perspective [6]. In order to study the structure, function and dynamics of directed PPI networks, multiple computational system biology approaches have been employed to reveal important links in various biological networks [7]. This includes, among others, finding physical interactions (e.g., between proteins in PPI networks) and functional interactions (e.g., between genes with similar or related functions, direct or indirect regulatory relationships between genes), identifying network modules (clusters of intensively interacting molecules) [7], interaction patterns and topological properties of disease networks (such as cancers, HIV infections, diabetes mellitus, Parkinson, Alzheimer, etc.) [8].

A number of computational pipelines and softwares have been developed [9] to perform various analysis of interaction patterns, topological properties, and visualisation of PPI networks. The majority of these approaches are focusing on finding structurally important disease-associated protein interactions in a network [10, 11]. However, so far there are no known software solutions analysing interaction networks for the purpose of identifying strategies to gain control over (parts of) the network. Recently, several algorithms have been developed to perform network structural analysis and suggesting optimal sets of so-called *driven* nodes through which one can control a network [12–14]. This paper aims to fill this gap by introducing the first open web-based tool implementing network controllability for biomedical networks.

A linear dynamical system is said to be *(fully) controllable* through a set of *driven nodes* if there exists a time-dependent sequence of input signals delivered through these nodes in such a way that, through cascading changes, the system can be driven from any initial state to any desired final state within finite time [12, 15]. In the biomedical domain, the interventions can be thought of as drugs delivered to a patient, and the driven nodes can be thought of as the drug targets. An efficient method to select a minimal set of driven nodes in *gene regulatory network* in order to reach its full controllability was recently presented in [12]. However, computer-based experimental tests in [12] shows that in biological networks one may have to control as much as 80% of the nodes of a gene-regulatory network in order to gain full controllability. This makes the full network controllability approach impractical for biological and medical purposes. In many cases, it is more practical to control only a certain subset of the network’s nodes (for instance, a disease-specific set of essential proteins) in order to reach a desired overall behavior of the system [13, 14, 16]. This approach, called *target controllability*, may lead, for instance, to realistic suggestions for combined multi-drug therapies for a particular disease [16]. We focus in this paper on target controllability.

We develop a bioinformatics data analysis pipeline (called *NetControl4BioMed*) and its web-based front-end in order to provide a web-based service for automatic generation of combined multi-drug therapy suggestions through the analysis of directed biochemical interaction networks. The pipeline generates automatically intracellular molecular interaction networks by combining the seed nodes provided by the user with interactions among proteins and other intracellular components from several public pathway repositories: KEGG, WikiPathways, and Pathway Commons. The core of the pipeline consists of the implementation of the algorithm proposed in [14]. For a given set of disease-specific essential proteins, the algorithm identifies in the network a small set of driven nodes through which one can gain control over the essential proteins. To boost the practical applicability of the pipeline, we implemented a version of the algorithm that uses data from DrugBank to maximize the use of drug-targetable proteins as driven nodes. The pipeline can be accessed and downloaded from [17].

## Methods

### Structural network control

We give a brief presentation of the network controllability approach and of the algorithm proposed for it in [14]. This algorithm aims to find a small set of driven nodes that can be used to control a given set of target nodes. The algorithm uses several heuristic strategies for an efficient exploration of the search space, which leads to faster and better (smaller sets of driven nodes) results in comparison to the original version of the target controllability algorithm proposed in [16].

We denote by **N** the set of nonnegative integers and by **R** the set of real numbers.

We consider discrete time-invariant linear dynamical systems as models of biological entities (proteins) influencing each other. Such a dynamical system describes a network where nodes influence each other’s evolution, while the time-invariant attribute establishes that these influences of the nodes over each other is not time dependent. Moreover, a number of external, so-called *driver* nodes are also connected to some of the internal nodes of the network and have a direct influence over their evolution. The model also includes the possibility of having a number of *output nodes* reflecting the evolution of the internal nodes of the network. A quantitative model can be associated to such a linear dynamical system by

$$x_{t+1}=Ax_{t}+Bu_{t}, y_{t}=Cx_{t}, $$

where *A*,*B*,*C* are matrices of size *n*×*n*, *n*×*m*, and *l*×*n*, respectively, *x*_*t*_∈**R**^*n*^, *u*_*t*_∈**R**^*m*^ and *y*_*t*_∈**R**^*l*^ are the state vector, input vector and output vector, for all *t*∈**N**. The state vector collects the configuration of the model at time *t* and has an entry for each node in the network. The input vector has an entry for each of the driver nodes and the output vector has one for each of the output nodes. Matrix *A* describes the interactions *within* the system under scrutiny; the entry *a*_*i*,*j*_ of matrix *A* describes the weight of the influence of node *j* over node *i*. As the graph is directed, the system is in general asymmetric: the influence of node *i* over node *j* need not be equal with the influence of node *j* over node *i*. Matrix *B* describes the influence of the *m* driver nodes over the internal nodes of the system, while *C* describes the *l* output nodes as a function of the internal nodes of the system. We call *driven node* any *i*∈{1,…,*n*} such that *B*_*ij*_≠0, for some *j*∈{1,…,*m*}; in other words a driven node is any internal node linked to an external driver node through matrix *B*. We say that an output vector *y*∈**R**^*l*^ is *reachable* from an initial state *x*_0_∈**R**^*n*^ if there exists a finite sequence of inputs *u*_0_,*u*_1_,…,*u*_*t*_∈**R**^*m*^ such that *y*_*t*_=*y*.

**Fig. 1:**
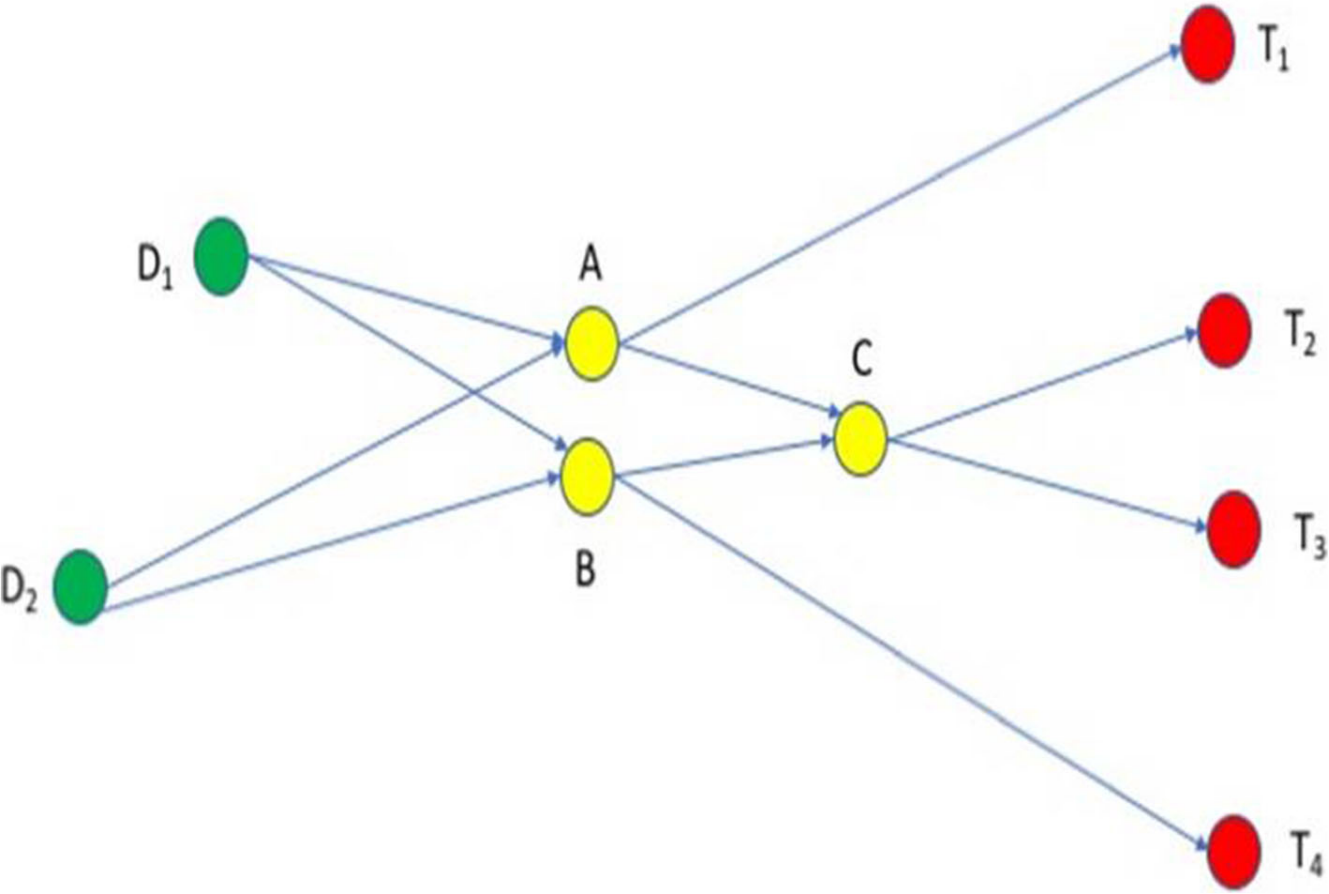
Targeted structural controlability. The targeted structural controllability problem for the directed graph *G*=(*V*,*E*) with *n* nodes and a subset *T*⊆*V* with *I* target nodes, is equivalent with deciding if there exists a set of *l* directed paths in *G* such that each node in *T* is an end point of one such path and no two paths intersect at the same distance from their end points, [15]. In this example, the paths from the driven nodes *D*_1_, *D*_2_ to the target nodes *T*_1_−*T*_4_ intersect in the internal nodes *A*, *B*, and *C*. The controllability theorem of [15] implies that the lengths of the paths *CT*_2_ and *CT*_3_ is different, and that either the length of the path *AT*_1_, *AT*_2_, and *AT*_3_ are pairwise different, or the length of the path *B*_*T*_2, *B*_*T*_3, and *BT*_4_ are pair-wise different (or both)

In this paper we focus on target controllability, i.e., on the case where the aim is to control a well-defined subset of the internal nodes of the system. To capture this case, we consider matrices *C* with *l*≤*n* and such that on each row of matrix *C* there is at most one non-zero value; this effectively selects the internal nodes of interest as outputs of the dynamical system. We say that such a system is *target controllable* if any output vector is reachable from any input state. It is known that a system is target controllable if and only if

$$rank\left[CB, CAB, CA^{2}B, \ldots, CA^{n-1}B\right]=l, $$

see [14] and references therein. A related notion is that of *structural target controllability*, that refers to a system that becomes target controllable by changing the non-zero values of *A* and *B* with some well-chosen non-zero values (we call such matrices *equivalent*). The difference between target controllability and structural target controllability is significant: in the former case the precise numerical setup of the network is crucial for the controllability of the network, whereas in the latter case only the structure is of interest, not the numerical setup. The focus on the structural (target) controllability is justified by the difficulty to measure *precisely* numerical parameters, and by the many numerical parameters left unmeasured in large network models. The question for structural controllability thus is: given a network of interactions, does there exist *any* numerical setup that may make it controllable? The freedom in choosing any numerical setup does not hamper the practical applicability of this approach to a specific case, where the numerical setup is fixed. Indeed, a deep result of [15, 18] shows that a system is structurally target controllable if and only if it is target controllable for all equivalent matrices *A* and *B*, except a so-called “thin” set of matrices. (It is beyond the goal of this paper to define the topological notion of thin sets; we only give here the intuition that such sets consist of isolated cases that may be easily replaced with nearby favourable cases.) The benefit of this result is that by focusing on structure rather than on highly precise numerical setups, the problem becomes one on directed graphs, rather than on algebra. For details we refer to [14] and references therein. We only mention here that the problem may be formulated on directed graphs as follows: given a directed graph *G*=(*V*,*E*) with *n* nodes and a subset *T*⊆*V* with *l* nodes, decide if there exists a set of *l* directed paths in *G* such that each node in *T* is an end point of one such path and no two paths intersect at the same distance from their end points, see [15] and Fig. 1. In an additionally constrained version of the problem, one may also be given a subset *D*⊆*V* (e.g., corresponding to known drug-targets) and require that the directed paths preferably start from nodes in *D*.

The targeted structural controllability was proved to be computationally highly difficult in [14], where it was shown to be NP-hard. This means that calculating the minimal (in the sense of smallest) set of driven nodes to control a given set of targets is exponential in the size of the network, and thus unfeasible for practical real-life case studies. Instead, the authors in [16] proposed heuristics for giving some set of driven nodes, hopefully small, and in any case not guaranteed to be minimal. In [14] faster algorithms were proposed, based on stochastic searches for paths to the target nodes. These algorithms remain approximation heuristics and give no guarantee that they will find a minimal set of driven nodes; in the tests we made they returned results that are a degree of magnitude smaller than those in [16]. The implementation we chose for them in our pipeline is based on thousands of independent runs of the algorithm, with the best of the results reported as the final result.

## Implementation

Here we discuss the software tools used to build our pipeline and the data used in it.

### Workflow engine: Anduril

The pipeline is developed for the *Anduril* workflow framework [19]. *Anduril* is an open source component-based pipeline engine for scientific data analysis. Anduril defines an API (Application programming interface) that allows to integrate rapidly a vast range of existing software analysis and simulation tools and algorithms into a single data analysis pipeline. An *Anduril* pipeline represents a set of interconnected executable programs (called components) through well-defined I/O ports. Upon the termination of the execution of an *Anduril* component, its output results are delivered as inputs to the other (downstream) components by means of connecting the output port of the component to the input ports of its downstream components. When an Anduril pipeline is being executed, a component can be executed as soon as all the necessary input data at the input ports (from the upstream components) become available.

#### Biological data and network generation

Our pipeline uses the *Moksiskaan* platform [20] to generate molecular interaction networks based on the user’s query. Moksiskaan integrates pathways, protein-protein interactions, genome and literature mining data into comprehensive networks, starting from a given list of proteins (so-called “seed nodes”). It combines the relations among proteins from different known pathways in order to address the fact that pathways crosstalk and influence each other. The Moksiskaan platform defines a generic database schema to store the pathways from a number of different pathway databases and can be scaled to include the pathway data from new sources (such as new databases and user’s own data). Currently, Moksiskaan has built-in support for the integration of the pathway data from, among others, KEGG pathway database [21], Pathway Commons [22], and WikiPathways [23, 24].

In our pipeline, Moksiskaan constructs a comprehensive network for the list of seed nodes by using and combining all imported pathways in the following manner: it connects all seed nodes by all known paths of length not exceeding the “gap” value. The gap, a parameter that the user may set in the pipeline GUI, is the maximum number of intermediate nodes the network may have between the seed nodes. For higher gap values, the network will grow quickly in size as the pipeline will search for any paths of length up to gap+1 between the seed nodes, and add them to the network, along with all the intermediary nodes. The higher the gap, the more comprehensive the network will be and the smaller the set of identified driven nodes will be, but also the slower the network analysis will become. The pipeline currently includes the option of selecting a gap value up to 5.

We use drug-target protein data from the open source DrugBank database [25]. The DrugBank database combines detailed drug (i.e. chemical, pharmacological and pharmaceutical) data with comprehensive drug-target (i.e. sequence, structure, and pathway) information from bioinformatics and cheminformatics resources. For drug-target identifiers we selected all FDA (Food and Drug Administration)-approved drug-target proteins with known mechanisms, in total 1507 proteins.

We provide the user with a number of predefined sets of target proteins associated to some specific cancer cell lines. These target proteins are cancer-specific essential proteins. We have included in the pipeline data for three types of cancer after mapping from the COLT-Cancer database [26]. In particular, we considered 29, 23 and 15 cell lines respectively for breast, pancreatic and ovarian cancer. Previous studies [27] showed that proteins with lower GARP (Gene Activity Rank Profile) score are stronger associated with oncogenesis. Therefore, we have selected only those essential proteins whose GARP value is in the negative range, and whose GARP-P value is less than 0.05. For more details about calculating GARP score, see [26].

### Pipeline structure

**Fig. 2:**
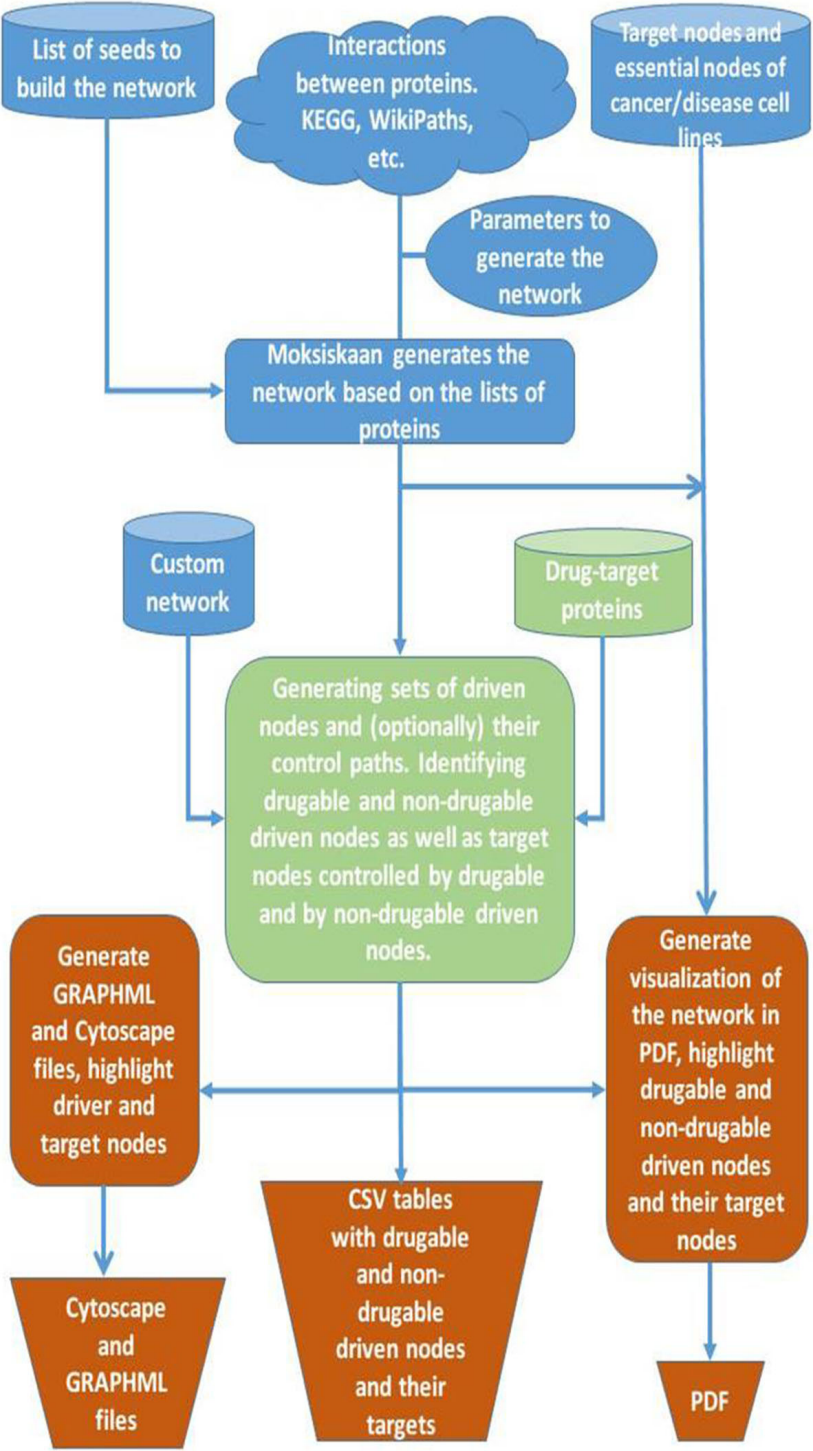
The general scheme of the *NetControl4BioMed* pipeline. The pipeline consists of three parts. In the first part we perform data input and preprocessing: we get from the user the list of seed nodes, the predefined list of essential proteins, and the list of additional target nodes, if provided by the user. Moksiskaan generates the network based on the seed proteins provided by the user; the seed can also include the predefined list of cancer cell line-specific essential proteins and the optional list of user-defined target nodes. The user also can provide for the analysis a custom network instead of that generated by Moksiskaan. The second part of the pipeline deals with the network structural controllability analysis, where a minimal set of driven nodes is computed for the given set of target nodes (user-defined target nodes and cancer cell line-associated essential proteins). In the third part of the pipeline the post-processing is performed and the output is generated. In the output, the user gets the network generated by Moksiskaan and the information about driven nodes, target nodes and drug-targetable driven nodes

Here we describe the pipeline structure as well as its input and output, see Fig. 2.

### INPUT

Our pipeline currently accepts the following inputs from the user:

1. **Seed proteins**: List of proteins that will be used as seed nodes by Moksiskaan to generate the network. This input can be any protein ID of Homo sapiens.
2. **User-defined network**: The user has the option to use a custom network in the pipeline instead of the Moksiskaan network.
3. **Cancer Cell Lines**: The user has the option to include data on a cancer cell line, whose set of essential proteins will be used as target nodes and/or as seed nodes. If the user does not include any cancer line, then the next field should not be empty.
4. **Additional target proteins**: A set of target nodes defined in addition to those in the “Cancer Cell Lines”. This input can be left empty if the previous field is set to a cancer cell line. These nodes may also be included as seed nodes.
5. **Gap**: The gap parameter used by Moksiskaan to generate the network.
6. **Include drug information**: This is an option on whether the pipeline should include also the drug-target information for the driven nodes. If so, then the driven nodes for which there exist FDA approved drugs will be specifically highlighted in the output of the pipeline.
7. **User defined drug-target proteins to be included in the analysis**: The user has an option to include also set of custom drug-target proteins. If the “Target By Drug” field is chosen, the user-defined custom drug-targets will be considered along with the FDA-approved drugs-targets.

### OUTPUT

**Fig. 3:**
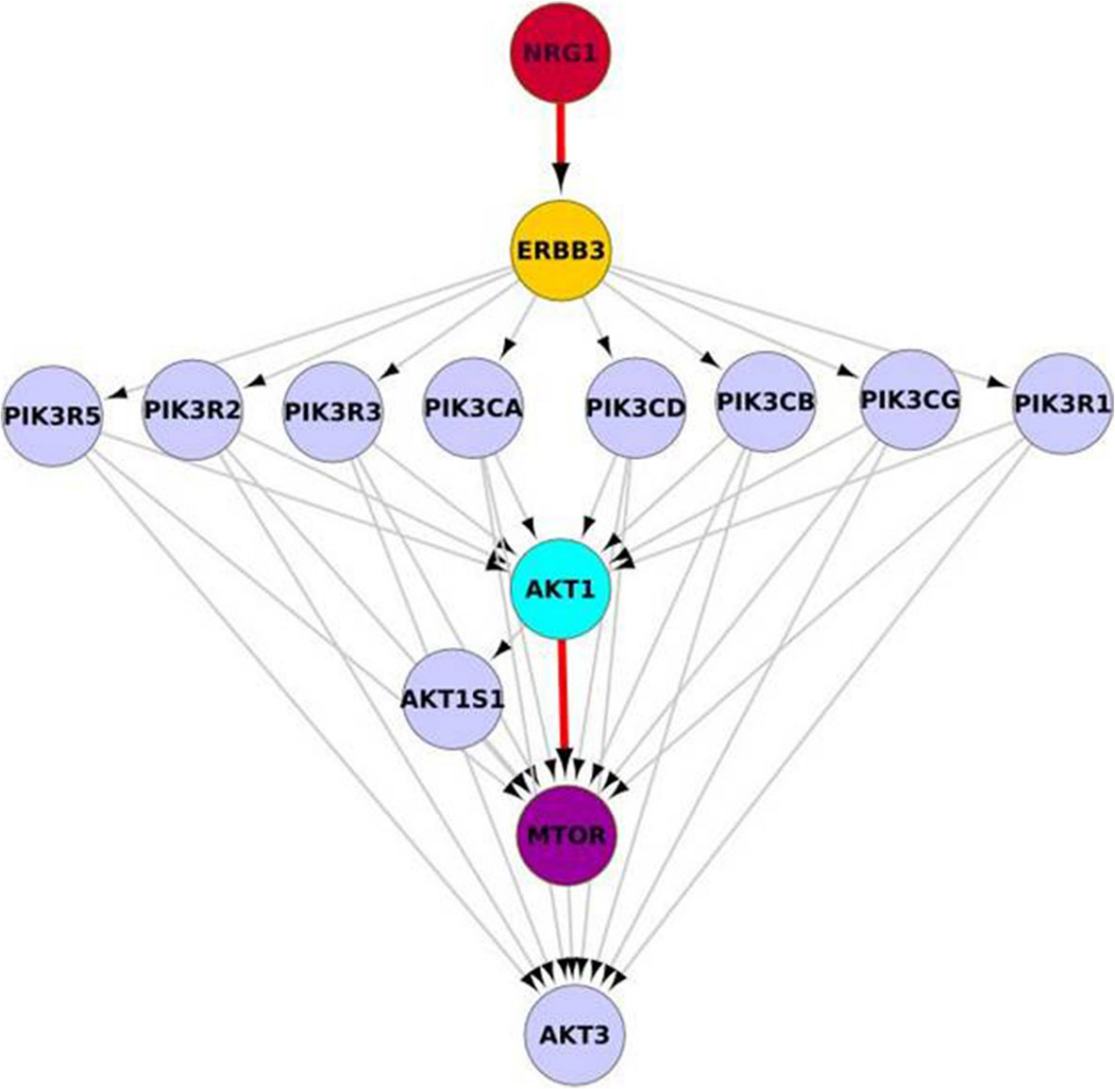
A visualization of the generated network from the pipeline. Proteins PIK3R3, PIK3CB, PIK3R1, PIK3CG, PIK3CD, PIK3CA, PIK3R5 and PIK3R2 are promoted/activated by ERBB3. They promote/activate AKT1, AKT2, AKT3 and MTOR and inhibit AKT1, AKT2 and AKT3. Proteins PIK3R3, PIK3CB, PIK3R1, PIK3CG, PIK3CD, PIK3CA, PIK3R5 and PIK3R2 have no interactions between each other. NRG1 controls ERBB3 and AKT1 controls MTOR. The colors have the following meaning: *“seed nodes”* are shown in green circle (NRG1, ERBB3, MTOR), *“driven drug-target nodes”* are represented as aqua color (AKT1), *“controlled from drug-target nodes”* are shown in purple color (MTOR), *“driven non-drug-target nodes”* are shown in red color (NRG1) and *“controlled from non-drug-target nodes”* are shown in orange yellow (ERBB3)

The heuristics used for the target controllability algorithms are stochastic, see [14]. This means that for the same input, different outputs may be generated. The pipeline generates as the result of the computation a *zip-*archive with the following files. Table *driven.csv* contains the drug-targetable driven nodes and the number of targets (e.g., cancer essential proteins) controlled by them. File *driven.csv* will be empty if no target could be found that can be controlled by the drug-target driven protein. Table *extra.csv* contains the non-drug-targetable driven nodes (no FDA-approved drug-target proteins are known to be targeting the node) and the number of targets (e.g., cancer essential proteins) controlled by them. File *extra.csv* will be empty if no target could be found that can be controlled from a non-drug-targetable driven protein. In *details.txt* the first line indicates the heuristics which was used for obtaining the result in the file. A blank line follows, then the names of the driven nodes, each on a separate line. After another blank line, it shows the entire (control) path of targeted nodes in the network from the driver nodes. File *graph.xml* contains the generated network and can be visualized in *Cytoscape* and further downloaded as a *node.csv* from *Cytoscape*. The archive also contains a visualization of the controlled graph (as a PDF file) generated with GraphML, see Fig. 3.

## Results and discussion

The network in Fig. 3 is generated based on breast cancer specific proteins. Here, we selected the *AKT1, AKT3, NRG1, MTOR, ERBB3* protein as seed nodes to generate the network. We chose *MTOR* and *ERBB3* proteins as target proteins, as we found these as essential proteins in cancer cell lines MBD-MB-231. Here, *AKT1* is a drug-targetable driven node through which control can be gained over the cancer essential protein *MTOR*. Dysregulation of *MTOR* pathways lead to oncogenesis in breast cancer [28]. It has been seen that HER2 over-expression by *MTOR* is one of the main cause of breast cancer [29, 30]. It has also been shown that *AKT* is one of the critical anticancer drug-targets for rational drug discovery being present as a site in various multiple oncogene and tumor suppressor signaling networks [31]. The non-drug-targetable node *NRG1* is also predicted by our algorithm to be able to gain control over cancer essential protein *ERBB3*. *NRG1* is known to be involved in the dysregulation of *ERBB3* (*ERBB3* has prominent role in oncogenesis) [32, 33].

To demonstrate the wide applicability of the pipeline and its algorithmic back-engine, we also analyzed two case studies on Type 2 diabetes and on Alzheimer disease protein-protein interaction networks. For Type 2 diabetes we gathered literature data on essential proteins from [34–37]. Alzheimer’s essential protein data was gathered from [38–42].

In the case study on the Alzheimer disease, our pipeline reported *MTOR* as a driven node through which control can be gained over the essential protein *NOS3*, see (Additional file 1: Figure S1). *NOS3* is well known for its association with *G894T* as a main risk factor of Alzheimer’s disease [43, 44]. Previous research shows that MTOR could be a remarkable target for Alzheimer’s disease [45, 46]: the dysregulation of *MTOR* signaling pathway is involved in the pathogenesis and progression of Alzheimer’s Disease. Also, the use of *MTOR* inhibitors was reported as a therapeutic target for Alzheimer’s disease in [47].

In Type 2 diabetes, our pipeline reported *MYC* as a driven node through which control can be gained over the essential protein *CDKNB2* see (Additional file 1 Figure S2). This result correlates with earlier predictions of *MYC* as drug-target in various cancers [34]; interestingly, *MYC* is not yet documented to be used in treatment options for Type 2 diabetes. With SNPs in their 3’ UTR miRNA binding sites, *CDKN2B* increase the risk phenotype. Further, pancreatic beta-cell replication is regulated by *CDKNB2* [48] and its faulty regulations increase the risk of diabetes.

The structural network controllability approach allows to get a better insight into a system modeled as a directed graph: for a set of target nodes it is possible to identify a set of driven nodes through which one can control the target nodes by an external intervention through using the internal “wiring” of the network. It is a promising approach that allows one to design a system-level handle into directing the evolution of a complex system. Moreover, the approach even allows the modeler to focus on the structure of the network, while avoiding the need to measure or identify many numerical parameters. It is widely applicable to any model presented as a directed network, with a set of key nodes whose indirect control is to be gained. Signalling transduction networks are particularly suitable for this approach. Other types of networks, e.g., metabolic networks, remain outside the applicability domain of this approach, as they are not amenable to being modeled as directed graphs.

We use here a recently developed algorithm [14] for structural targeted network controllability that identifies a minimal set of driven nodes for a user-given set of target nodes. We implemented this algorithm through a pipeline (that can be downloaded and installed as a stand-alone software) and through a related online service (a publicly available web interface for an instance of the pipeline installed on our servers). The pipeline performs an automatic generation of intracellular molecular interaction networks (by combining publicly available pathway data) and identification of driven nodes (which also can be targeted by FDA approved drug target-proteins) for a set of target proteins defined by the user.

In this paper we also address the interesting problem of using the controllability approach for a combination of data on FDA-approved drug-targets and data on cancer essential proteins for different types of cancers. Users can also apply this pipeline if they have other disease-specific target proteins. We anticipate that our pipeline has the potential in suggesting novel therapeutic strategies by using currently known drugs.

The benchmark tests have shown the following results for our pipeline. When using under 10 seed nodes and gap 1, the pipeline generates networks of a size close to 30 nodes and 100 edges (the exact values depend on what seed nodes have been chosen exactly and what interactions between the nodes are known in the databases). Our structural network controllability algorithm processes networks of this scale and finds the driven nodes (in the pipeline GUI called *input nodes*) in time of 1 second. For 10 seed nodes and gap 2 the pipeline generates networks in range 20 to 50 nodes and 30 to 300 edges. Networks of this scale are being analyzed by our algorithm in range of 1 to 3 seconds. When used near 20 seeds and gap 1 or under 10 seeds and gap 3, the pipeline generates networks of size close to 100 nodes and 1.000 edges. The algorithm analyzes the networks of this size in 5 seconds. If using near 20 seeds and gap 2, we get networks near 200 nodes and 2.500 edges. The analysis runs here near 20 seconds. For 20 nodes with gap 3 and 4 we get networks from 300 to 600 nodes and 6.000 to 9.000 edges. The analysis takes here from 30 to 50 minutes. The pipeline generates networks with near 800 nodes and 11.000 edges for near 20 seeds with gap 5. The algorithm computes driven nodes for this network in near 7 hours.

Hereby, we conclude that our pipeline is practical for analysis of networks of size up 1.000 nodes and 10.000 edges, since the results can be obtained within 1 day. For small networks (up to one hundred nodes and 2.000 edges) the result is obtained in time up to 2 minutes. We note that in practice the computational time needed for the algorithm starts growing extremely fast when approaching size of 3.000 nodes in a network. Also, the efficiency of the pipeline strongly depends on how many free CPU cores the host system provides, since the python implementation of our network target controllability algorithm relies heavily on usage of parallel threads. In particular, we have been running several computationally heavy pipeline tasks on a single system with 12 free CPU cores while performing the benchmarking for this article.

The pipeline can be accessed and downloaded from [17].

## Conclusion

The software we discussed in this article opens up the network controllability methods for applications in a variety of domains. The focus has been on a user-friendly interface that includes a text-based input, a visual output, output files that are compatible with standard modelling software, web-based interface requiring no special installations on the user’s end. There is extra support offered by the software for users in cancer medicine in the pre-loaded list of essential genes in several types of cancer. We believe that the pipeline can be used by researchers for controlling and better understanding of molecular interaction networks through combinatorial multi-drug therapies, for more efficient therapeutic approaches and personalised medicine.

## Availability and requirements

**Project home page:**http://combio.abo.fi/research/network-controlability-project/**Operating system(s):** Platform independent, browser-based.**Programming language:** Anduril, Python, PHP.**Other requirements:** Modern webbrowser.**License:** FreeBSD.**Any restrictions to use by non-academics:** none.

## Additional file

Additional file 1 Three examples – breast cancer, diabetes, and Alzheimer’s disease. (PDF 1030 kb)
