# Supplementary material for: NetControl4BioMed: a pipeline for biomedical data acquisition and analysis of network controllability
Source: BMC Bioinformatics. 2018 Jul 9;19(Suppl 7):185. doi: 10.1186/s12859-018-2177-3 (PMC6069765; doi:10.1186/s12859-018-2177-3)
Supplement: Supplementary file 1 — Three examples – breast cancer, diabetes, and Alzheimer’s disease. (PDF 1030 kb) [file 12859_2018_2177_MOESM1_ESM.pdf]

Additional file 1: Three examples – breast cancer, diabetes, and Alzheimer's disease.

Breast cancer

The breast cancer PPI network is found as Figure 3 in main manuscript.

Output table of breast cancer:

driven.csv	extra.csv	details.csv
AKT1	NRG1	NRG1<br>AKT1<br><br>ERBB3 <- NRG1<br>MTOR <- AKT1

Diabetes

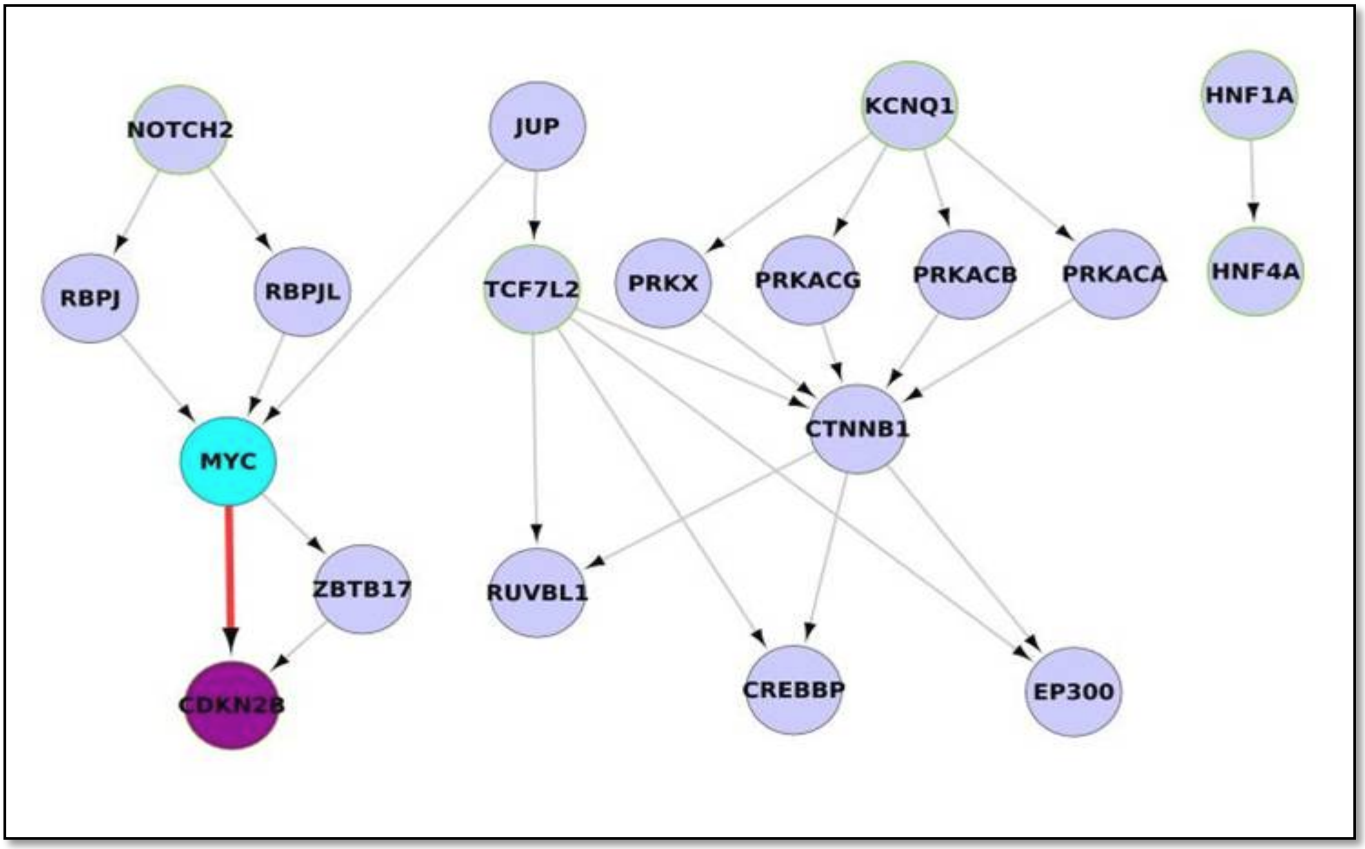

Fig S1. Diabetes PPI network. A network view of the diabetes PPI network. CDKNB2 are prompted/activated by ZBTB17. Proteins MYC is drug-target protein through which control can be gained over the essential protein

CDKNB2. The colors have the following meaning: “seed nodes” are shown in green circle, “driven drug-target nodes” are represented as aqua color (MYC), “controlled from drug-target nodes” are shown in purple color (CDKN2B).

Output table of diabetes:

driven.csv	extra.csv	details.csv
MYC		CDKN2B <- MYC

Alzheimer’s disease

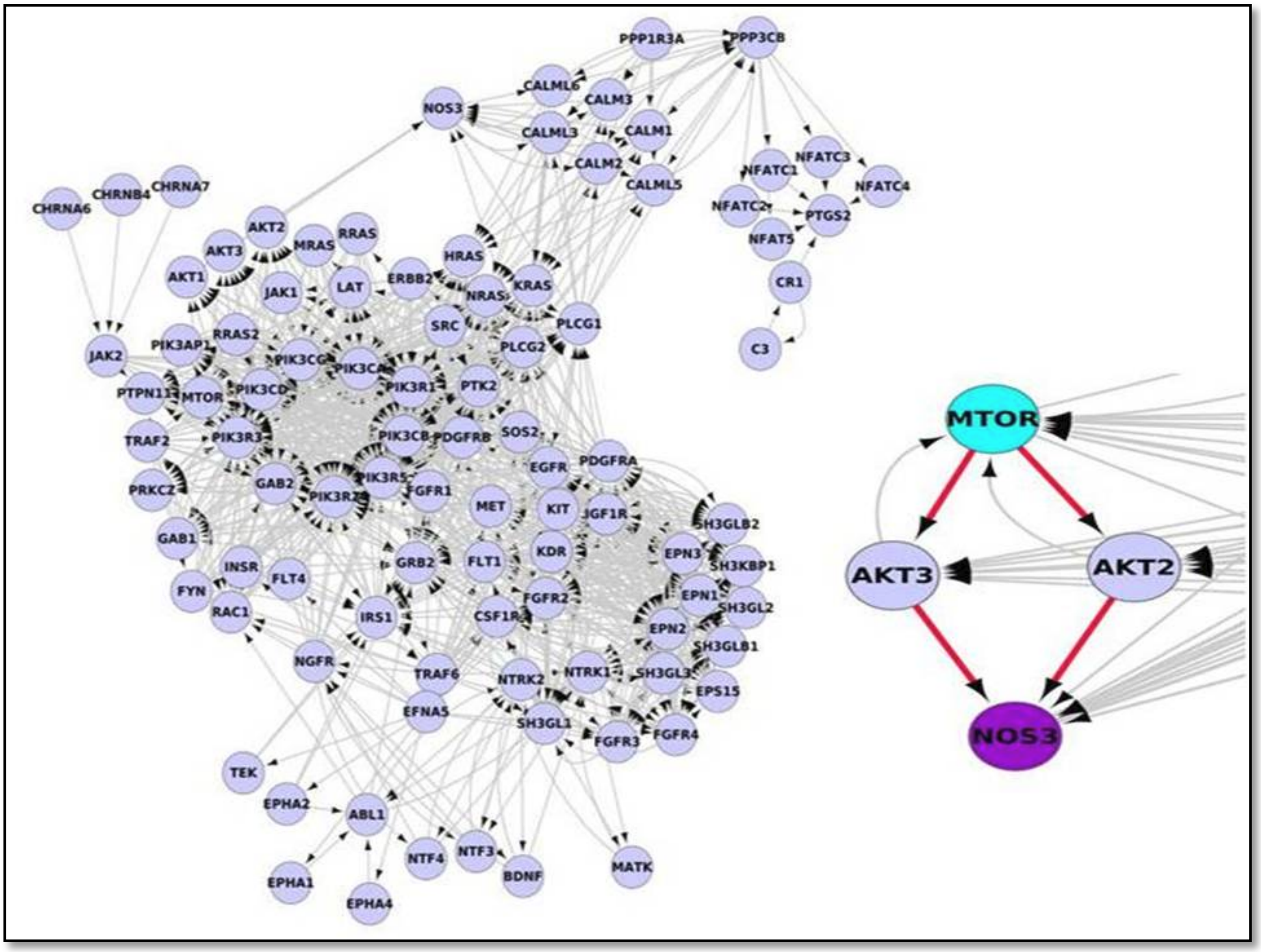

Fig S2. Alzheimer’s disease PPI network. A network view of the Alzheimer’s disease PPI network. NOS3 are mainly prompted/activated by AKT2/AKT3. MTOR is a drug-target protein through which control can be gained over the essential gene NOS3. NOS3 is well known for its association with G894T as a main risk factor of Alzheimer’s disease. The colors have the following meaning: “seed nodes” are shown in green circle, “driven

drug-target nodes” are represented as aqua color (MTOR), “controlled from drug-target nodes” are shown in purple color (NOS3).

Output table of Alzheimer’s disease:

driven.csv	extra.csv	details.csv
AKT1		AKT1<br><br>NOS3 <- AKT1 <- MTOR
